# Combination of Antiretroviral Drugs Zidovudine and Efavirenz Impairs Tumor Growths in a Mouse Model of Cancer

**DOI:** 10.3390/v13122396

**Published:** 2021-11-30

**Authors:** Marcel A. Schneider, Anton A. Buzdin, Achim Weber, Pierre-Alain Clavien, Pieter Borger

**Affiliations:** 1 Laboratory of the Swiss Hepato-Pancreato-Biliary (HPB) and Transplantation Center, Department of Surgery, University Hospital Zürich, Raemistrasse 100, CH-8091 Zürich, Switzerland; marcel.schneider@uzh.ch (M.A.S.); clavien@access.uzh.ch (P.-A.C.); 2 Moscow Institute of Physics and Technology, Dolgoprudny, 141701 Moscow, Russia; bu3din@mail.ru; 3 World-Class Research Center “Digital Biodesign and Personalized Healthcare”, Sechenov First Moscow State Medical University, 119991 Moscow, Russia; 4 Omicsway Corp., Walnut, CA 91789, USA; 5 Shemyakin-Ovchinnikov Institute of Bioorganic Chemistry, 117997 Moscow, Russia; 6 Institute for Pathology, University Hospital Zürich, Raemistrasse 100, CH-8091 Zürich, Switzerland; achim.weber@usz.ch

**Keywords:** combination therapy, antiretroviral drugs, zidovudine, efavirenz, cancer treatment

## Abstract

LINE1 retrotransposons, which are thought to be the remnants of ancient integrations of retrovirus-like elements, are aberrantly (re)activated in many cancer cells. Due to LINE1-induced alterations in target gene expression and/or chromosomal rearrangements, they may be important drivers of tumorigenesis. Moreover, LINE1 encoded proteins, Open Reading Frame (ORF)1 and ORF2, may have pro-oncogenic potential through inductors of oncogenic transcription factors or inhibitors of cell cycle suppressors. The current study therefore aimed to investigate in vitro and in vivo anti-tumorigenic effects of two well-known antiretroviral drugs, zidovudine, a nucleoside analogue inhibitor of RT (NRTI), and efavirenz, a non-nucleoside RT inhibitor (NNRTI). Our data demonstrate that both drugs in clinically relevant doses significantly reduced the proliferation of murine and human cancer cell lines, as well as growth of tumors in a murine subcutaneous model. Intriguingly, we found that the combination of both zidovudine and efavirenz almost entirely blocked tumorigenesis in vivo. Because both drugs are FDA-approved agents and the combination was very well tolerated in mice, the combination therapy as presented in our paper might be an opportunity to treat colorectal tumors and metastasis to the liver in an inexpensive way.

## 1. Introduction

Endogenous Long INterspersed Elements (LINE), which belong to the superfamily of transposable genetic elements, are a major part of higher eukaryotic genomes. In the human genome, LINE1 is the only active autonomous transposable element and with around 900,000 copies makes up 19.6% of the entire human DNA [1]. Although their biological function is largely unknown, the tightly controlled transient expression of LINE1 is associated with early stages of germ cell and embryonic development [2]. In cancer cells, however, the endogenous LINE1 machinery is aberrantly (re)activated [3]. During tumorigenesis, LINE1 exerts both retrotransposition-dependent and independent functions. The former may result in alterations of target gene expression or chromosomal rearrangement, events that could have functional roles in tumorigenesis. Moreover, the LINE1 encoded proteins, Open Reading Frame (ORF)1 and ORF2, may have pro-oncogenic potential through induction of oncogenic transcription factors or by inhibiting tumor suppressor genes. Indeed, several reports show that the ORF2, which encodes the endogenous reverse transcriptase (RT), plays a key role in tumorigenesis [4,5,6]. In turn, downregulation of LINE1 expression by transient RNA interference (RNAi) was enough to reduce tumor cell proliferation [7]. Furthermore, LINE1 activity differs among and within cancer types and fluctuates during cancer progression and evolution [8,9]. For instance, somatic LINE1 insertions are frequently found in colorectal cancer (CRC), less abundant in prostate and ovarian cancers, and extremely rare in multiple myeloma and glioblastoma [10].

Many studies have investigated the associations between LINE1 methylation levels, LINE1 transcriptional activity and cancer risk, progression, and prognosis, with most of them supporting correlations between tissue LINE1 hypomethylation and increased cancer risk or poor prognosis [8,11,12,13]. Importantly, LINE1 hypomethylation correlated with unfavorable prognosis of many cancers, including CRC [14] and hepatocellular carcinoma (HCC) [15]. Moreover, the majority of invasive breast cancers expressed LINE1 proteins in the cytoplasm, with 28–31% of them showing nuclear expression [16]. In summary, LINE1 activity is increased in cancers and correlates with a poor prognosis. If LINE1 reactivation is important for tumorigenesis and/or metastasis, then it might be possible to influence these processes using specific inhibitors of LINE1-encoded reverse transcriptase (RT).

Sciamanna and coworkers proposed a model whereby LINE1-derived RT is critically involved in cancer development by driving a so far unknown global regulatory process connected with cell transformation and tumorigenesis. In vitro experiments demonstrated that nevirapine and efavirenz, two drugs widely used in AIDS treatment, inhibited cell proliferation and promoted differentiation of human tumorigenic cell lines [4,5]. Further, Dai et al. showed that the EC50 of efavirenz to inhibit LINE1 activity was around 10^−5^ M, a non-toxic concentration that can be reached by oral administration [6].

The current study therefore aimed to investigate in vitro and in vivo anti-tumorigenic effects of two well-known antiretroviral drugs, zidovudine, a nucleoside analogue inhibitor of RT (NRTI), and efavirenz, a non-nucleoside RT inhibitor (NNRTI). Our data demonstrate that both drugs in clinically relevant doses significantly reduced the proliferation of murine and human cancer cell lines, as well as growth of tumors in a murine subcutaneous model. Intriguingly, we found that the combination of both drugs almost entirely blocked tumorigenesis in vivo.

## 2. Methods

***Mice:*** All animal experiments and interventions were in accord with Swiss Federal Animal Regulations and approved by the Veterinary Office of Zürich (Approval Nr. 171/2017). Animals were kept on a 12 h/12 h day/night cycle with access to water and standard rodent chow ad-libitum. Female mice aged 8–10 weeks at the start of the experiment were used for all experiments. Wild-type mice (Wt, C57BL/6J) were obtained from Envigo (Horst, The Netherlands).

***Subcutaneous tumor cell injection:*** Subcutanous (S.c) tumor growth was initiated by injection of 2 × 10^5^ syngeneic colorectal tumor cells (MC38-GFP+) in 100 μL PBS at the right flank of mice. Length and width of tumors were measured every second day with an electronic caliper and tumor volume in mm^3^ calculated according to the ellipsoid formula 4/3π * length/2 * width/2 * width/2.

***Tumor tissue microarrays (TMA):*** TMA of 78 different tumor entities and corresponding normal tissue, 144 CRC tumor samples and 48 HCC samples of patients, which underwent biopsy or surgical resection at the Dept. of Surgery (UHZ) were obtained for assessment of LINE1 expression by immunohistochemistry. The clinical and histopathologic data of the retrospective cohort have been collected and documented.

***Immunohistochemistry and Antibodies:*** Immunohistochemistry was performed as described in the standardized Biorad protocol (https://www.bio-rad.com/webroot/web/pdf/lsr/literature/Bulletin_6376.pdf, accessed on 1 November 2021). In short, equal amounts of protein (20 µg/slot) were electrophoretically separated in a commercially available precast polyacrylamide gel (Biorad). Total protein transfer was achieved from the gel to the membrane by electroblotting (100 V, 120 min). Detection of the LINE1 proteins was performed using the LINE1 specific antibody of Santacruz (M300, SantaCruz; now discontinued), which targets an epitope at the C-terminus of mouse and human LINE1 proteins. The detected protein was of the expected size (around 150 kD). The translocation index for LINE1 proteins was calculated and presented as the fraction of nuclear presence divided by cytosolic presence.

***Cell lines:*** The human cancer cell lines HepG2 (HCC) and HT29 (CRC) were used for in vitro experiments, while murine colorectal carcinoma MC38 cells, syngeneic for C57BL/6 mice, were used for in vivo studies. Cells were cultured in Dulbecco’s Modified Eagle Medium (DMEM) supplemented with 10% fetal bovine serum (FBS, Gibco, Life Technologies, Zug, Switzerland) and 100 U/mL each of penicillin and streptomycin at 37 °C and 5% CO_2_. All cell lines tested negative for mycoplasma at culture onset (PCR Mycoplasma Test Kit; PromoCell, Heidelberg, Germany) and were split by 0.05% Trypsin-EDTA (Gibco, Life Technologies, Zug, Switzerland) detachment every 2–3 days. Cells between passage 2–3 after thawing were used for all in-vivo and in-vitro experiments.

***MTT-assay (3-(4,5-dimethylthiazol-2-yl)-2,5-diphenyltetrazolium bromide):*** 10^4^ cells were plated in complete medium into 12-well cell culture plates and allowed to adhere for 4 h. Cells were then either treated with control (PBS) or with zidovudine (20 microgram/mL), stavudine (20 microgram/mL), abacavir (20 microgram/mL), efavirenz (20 microgram/mL) or a combination of zidovudine and efavirenz. After 2, 4 and 7 days, the cells were lysed by acidified isopropanol, and mitochondrial activity was measured by photometry at wavelength of 570 nm. All drugs were dissolved in PBS.

***In vivo drug administration:*** Zidovudine (30 mg/kg) and efavirenz (20 mg/kg) were dissolved in PBS from stock solutions and consequently administered over drinking water at indicated concentrations, starting 7 days after tumor cell injection until the end of the experiment. Controls received PBS only. We made a stock solution of efavirenz in DMSO solution (20 mM). Final concentrations of DMSO in cultured cells never exceeded 0.2%.

***Statistical Analyses:*** Descriptive statistics served to summarize groups with data displayed as mean ± standard deviation (SD). Groups were compared by students *t*-test or 1-way or 2-way analysis of variance (ANOVA) comparing tumor volumes over time for different groups with post-hoc Tukey’s multiple comparison test as appropriate and significance set at *p* < 0.05. All statistical analyses and graphical representations were performed using *R* version 3.5.1.

## 3. Results

### 3.1. LINE1 Expression in TMAs

**Figure 1:**
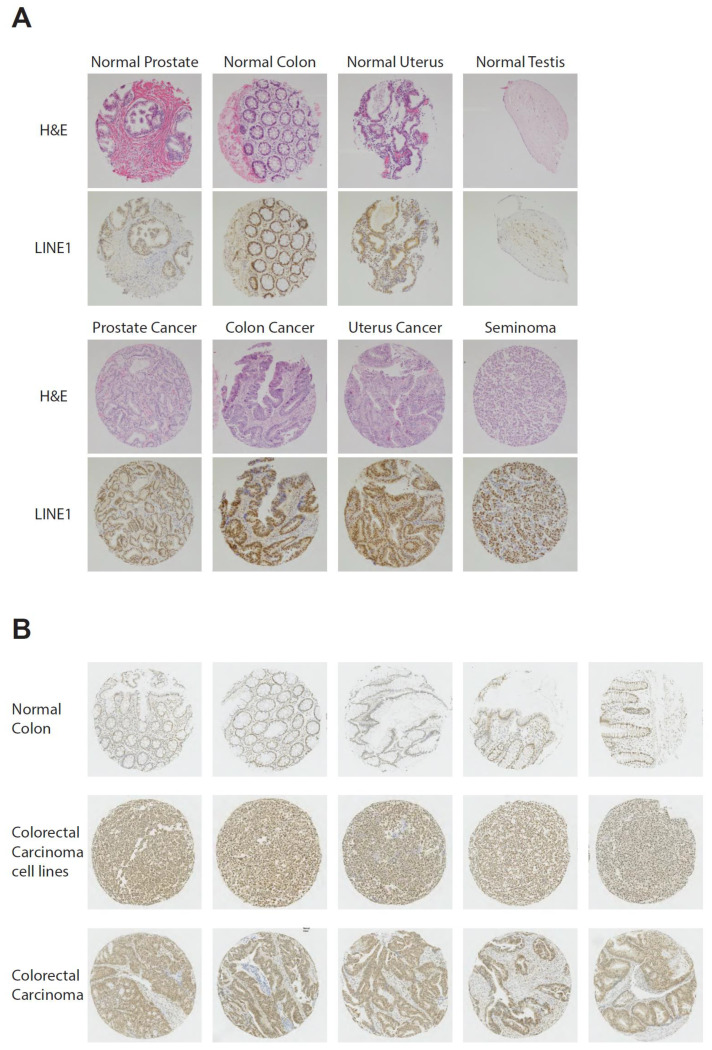
(**A**) Exemplary pictures of H&E and LINE1 immunohistochemical staining of normal tissues and malignant tumors, including prostate, colon, uterus and testis. (**B**) Exemplary immunohistochemistry of normal colonic tissue compared to colorectal carcinoma (CRC) cell lines and tissues of patients CRC. Note the increased LINE1 staining in all malignant tissue. All pictures taken at 10× magnification.

As a first step, we screened normal and pathologic human tissue samples on histological TMAs for LINE1 expression. On a first array including 78 samples of different human cancer types and corresponding normal tissues, a stronger immune-histochemical signal for LINE1 protein was observed for most cancer samples relative to normal controls (Figure 1A and Figure S1). For the current project, we consequently focused on CRC and HCC. Staining of a TMA panel with 144 CRC tissue samples detected LINE1 protein expression in 267 out of 288 tumors (92.7%) and 14 of 14 colorectal cancer cell lines (100%), while none of the normal colonic control tissue showed detectable signals (Figure 1B and Figure S2). Note that staining of tumor tissue was limited to adenocarcinoma cells, while cells of the tumor stroma showed little to no staining. This clearly indicates a specific signal in tumor cells only. In contrast, assessment of a TMA panel of human HCC samples, matching normal liver samples and benign tumor lesions did not demonstrate significant LINE1 staining of hepatocellular carcinoma cells (Figure S3).

### 3.2. LINE1 Expression Can Be Induced in HepG2 by PMA

**Figure 2:**
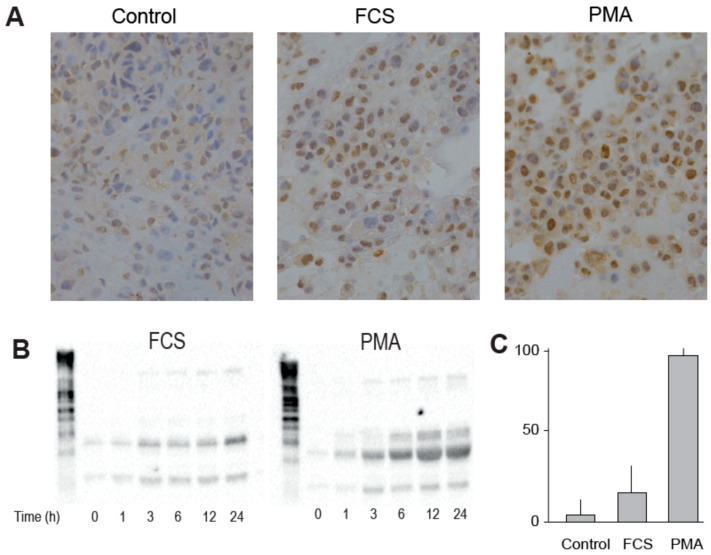
(**A**) Increased nuclear staining for LINE1 after treatment with FCS and PMA compared to untreated HepG2 cells. (**B**) Increased LINE1 protein expression as measured by western blotting. (**C**) Increased LINE1 translocation index as measured for nuclear presence of LINE1 protein.

We next tested if enhanced proliferation and metabolic activity in cancer cells were associated with an increase in LINE1 expression. The human HCC cell line HepG2 cultured in serum-free media expressed only marginal levels of LINE1 in the cytosol only. Mitogenic stimulation with fetal calf serum (FCS, 5%) resulted in 40–50% nuclear positivity of the Hep2G cells for LINE1, whereas after phorbol-myristate-acetate (PMA, 10 nM) treatment nearly ubiquitous LINE1 protein expression, predominantly in the nucleus, was observed in HepG2 cells (Figure 2A). Western blot analyses confirmed the lack of LINE1 protein expression in unstimulated HepG2 cells by western blotting. Both FCS and PMA dose-dependently induced high levels of LINE1 proteins, which peaked at >12 h (Figure 2B). Figure 2C shows the LINE1 translocation index, which is the measure of nuclear presence of LINE1 protein.

### 3.3. Effects of Reverse Transcriptase Inhibitors on Cell Metabolism/Proliferation

**Figure 3:**
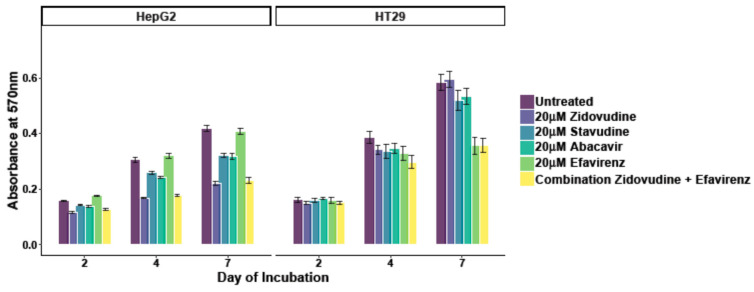
Decreased metabolic activity after treatment with Zidovudine in HepG2 cells and Efavirenz in HT29 cells after 2, 4 and 7 days of incubation.

Next, we tested the effects of four different antiretroviral drugs on proliferation and metabolic activity of human CRC (HT29) and HCC cell lines (HepG2,) using the MTT assay. The drugs had different effects on both cell lines. In HepG2 cells, zidovudine significantly reduced the metabolic activity (MA) by up to 47% by day 7 (*p* ≤ 0.001). Stavudine and abacavir reduced the MA by 23% (*p* ≤ 0.001) and 24% (*p* ≤ 0.001) at day 7, respectively. Efavirenz alone did not significantly affect the metabolic activity in HepG2 cells, neither did it show any synergistic effects in conjunction with zidovudine (*p* = 0.56, Figure 3). In HT29, the drugs did not show a significant effect after 4 days of incubation (zidovudine (3%, *p* = 0.63); stavudine (12%, *p* = 0.14); abacavir (13%, *p* = 0.07)) and did not further reduce MA after 7 days of treatment. After 7 days of treatment, only efavirenz (42%, *p* ≤ 0.001) and the combination of efavirenz plus zidovudine had a significant inhibitory effect (42%, *p* ≤ 0.001) on the metabolism of HT29 cells.

### 3.4. Effects of Zidovudine and Efavirenz on In Vivo Tumor Growth

**Figure 4:**
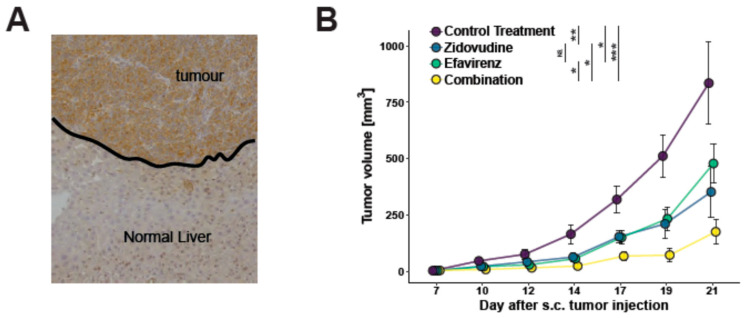
(**A**) Increased LINE1 staining of murine colorectal MC38 tumors growing as colorectal liver metastases compared to adjacent normal liver tissue. (**B**) Decreased tumor growth of s.c. injected MC38 cells in C57/Bl6 mice treated with either Zidovudin or Efavirenz. Note the synergistic effect of combination treatment resulting in strongly decreased tumor growth. * *p* < 0.05, ** *p* < 0.01, *** *p* < 0.005.

Based on the observed effects of reverse transcriptase inhibitors on in vitro cell growth reduction, we consequently aimed to test if these drugs could influence tumor growth in vivo. Immunohistochemical staining of pre-existing murine tumor samples showed high levels of LINE1 in the C57/Bl6 syngeneic colorectal tumor cell line MC38 growing in the liver after intraportal injection to mimic colorectal liver metastases, but not in the adjacent normal liver tissue (Figure 4A). We therefore chose to use this cell line for in vivo experiments. MC38 cells were injected subcutaneously in C57/Bl6 mice. The animals were randomized at day 7 according to the established tumor size into 4 groups of 5 mice each and consequently treated with either control treatment, zidovudine, efavirenz, or a combination of zidovudine plus efavirenz.

We noted significantly impaired tumor growth after administration of either zidovudine or efavirenz (Figure 4B). As shown in Figure 4B, the combination of both drugs together resulted in even more pronounced tumor size reduction than one of both monotherapies (Figure 4B), indicating that treatment with RTI can inhibit tumor growth in the clinical setting.

## 4. Discussion

The recent observation that LINE1 transcription is a wide-ranging mechanism to keep the chromatin accessible during early embryogenesis identified LINE1 transcription as a potential prerequisite and driving force for cell cycle progression and causative factor in tumorigenesis and metastasis [17]. Here, we describe for the first time that a combination of low (non-toxic) concentrations of nucleoside RT plus non-nucleoside RT inhibitors, efavirenz and zidovudine, significantly reduced tumor development in a s.c. model of cancer. The inhibitory effect of efavirenz on LINE1-derived RT and its anti-proliferative potential have been reported for breast [18] and pancreatic cancer cell lines [19]. Despite their inhibitory action on proliferating cell lines [6,18,19], antiretroviral agents have not yet been extensively tested in animal models of tumor development and progression. Using the MTT test, we observed that nucleoside RT inhibitors zidovudine and efavirenz, were able to significantly reduce the metabolic activity of both HepG2 and HT29 cells. For HepG2 cells, zidovudine was the most potent metabolic inhibitor, while the non-nucleoside RT inhibitor efavirenz did not affect the metabolic rate. In contrast, efavirenz inhibited cell proliferation better in HT29 cells. The nucleoside RT inhibitors predominantly act as competitive anti-substrates for the RT, inhibiting further reverse transcription. In contrast, non-nucleoside RT inhibitors act as non-competitive inhibitors. Together they exert a very potent inhibitory effect on tumor proliferation, with the combination of both drugs being able to almost block in vivo tumor growth completely, demonstrating their different mechanisms of action. It has been demonstrated that in vitro toxic EC50 of non-nucleoside RT inhibitors is very hard to achieve in patients. Only 1.5% of HIV patients taking these drugs reached the toxic EC50 [19]. However, this drawback may be overcome by the simultaneous administration of both non-nucleoside RT inhibitors and nucleoside inhibitors. Our study thus provides a rational for combination therapy, where much lower concentrations work additively or even synergistically. Our data also indicate that not all tumoricidal effects of non-nucleoside drugs—and additional potential anti-cancer drugs—can be studied using in vitro models. Furthermore, one can imagine combination therapies of RT inhibitors with conventional systemic drugs used for chemotherapy but in far lower concentrations than currently in use, thus avoiding or reducing severe side effects.

**Figure 5:**
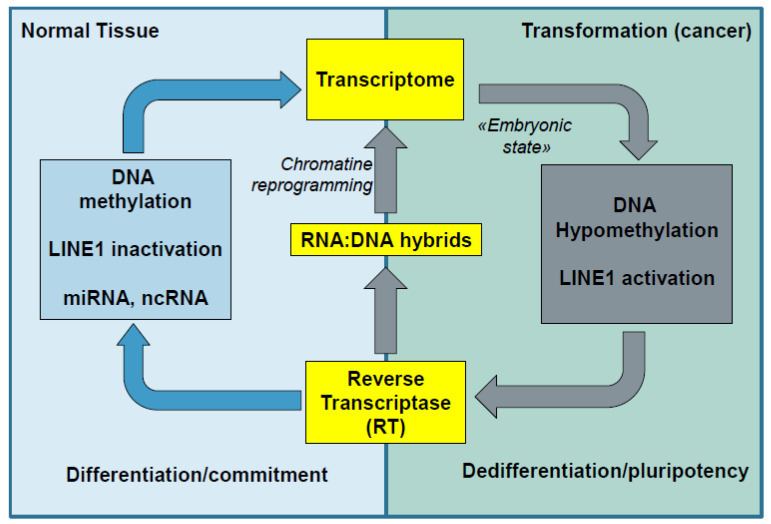
Model scheme of LINE1 working mode in normal cells and during malignant transformation. (Adapted from Sciamanna et al. [3]).

How and why LINE (re)activation occurs in cancer cells is largely unknown. We here showed that LINE1 expression is present in several malignant cell lines, which can be upregulated by additional mitogenic stimulation, such as FCS and PMA. After mitogenic stimulation, LINE1 protein is predominantly present in the nucleus. Despite the almost complete absence of LINE1 in unstimulated cultures of MC38 cells, we noticed significantly higher expression levels of LINE1 in MC38 tumors grown in vivo. LINE1 is thus an active part of the genome, which can be readily induced by unspecific cell mitogens, but is also present in developing liver tumors. Although evidence suggests that global hypomethylation involving LINE1 regions is an important cause of LINE1 reactivation in cancer, upstream regulators of this activation remain elusive. Some studies have suggested that both oxidative stress and interleukin-6 could be upstream reactivation signals—in particular in vivo [10]. Importantly, LINE ORF1 binds Smad4, thus keeping it in the cytosol unable to activate p21 gene expression [20]. Our hypothetical model of the action of LINE1, an adaptation of the model proposed by Sciammanna et al. [3], is demonstrated in Figure 5. Reactivation of LINE1 may thus counteract cell cycle silencing pathways, including those of the TGF family via the Smad-Foxo-p21 pathway, and re-establish an uncommitted or proliferative cellular phenotype through downregulation of Sirt6 and p21.

Earlier it was shown that downregulation of LINE1 expression by transient RNA interference (RNAi) also reduced cell proliferation and that the reverse transcription inhibitor abacavir showed anticancer activity in prostate cancer cell lines [21]. The changes in cell fate observed after inhibition of RT activity by either antiretroviral drugs or by RNAi were accompanied by a reprogramming of gene expression, possibly via LINE1 expression. Apparently, the endogenous RT protein emerges as a constitutive functional component in tumorigenic cells characterized by a high proliferation and a low differentiation status. Despite their inhibitory action on proliferating cell lines, antiretroviral agents have not yet been tested in animal models of tumor development and progression [7]. More recently, efavirenz has been tested in a phase II human trial on prostate metastatic patients [22]. In these studies, it was demonstrated that efavirenz alone did not statistically improve the PSA non-progression rate of prostate tumors. Nevertheless, their analysis suggested that higher plasma concentrations of efavirenz, i.e., the use of increased dosages, could be a potential treatment [22,23]. Our data suggest that not only increased dosages might be beneficial, but also combining two drugs with distinct molecular targets might improve treatment strategies. Similarly, Giovaninni et al. demonstrated that the responsiveness to antiretroviral drugs treatment of cancer cells with stemness features and expressing HERVs suggests the use of these drugs as an innovative approach to treat aggressive tumors in combination with chemotherapeutic/radiotherapy regimens [24].

In conclusion, we provide further evidence that the RT inhibitors zidovudine and efavirenz are able to impair cancer cell proliferation in vitro and in vivo, probably mediated by their effect on LINE1 transposition. Because both drugs are FDA-approved antiretroviral agents and the combination was very well tolerated in mice, we propose rapidly translating them clinically and testing them for their potential to be used as anti-tumorigenic drugs in humans. In addition to previous treatment strategies with antiretrovirals [25], the combination therapy described in our paper might help to further the fight against anomalous cell-proliferation disorders.

## Supplementary Materials

The following are available online at https://www.mdpi.com/article/10.3390/v13122396/s1, Figure S1: (A) H&E staining of complete tumor microarray of 78 different tumor entities. (B) LINE1 staining of complete tumor microarray of different tumor entities. (C) Detailed information of tumor entities. Figure S2: (A) LINE1 staining of complete tumor microarray of 144 patients with colorectal carcinoma, colorectal cell lines and normal colonic tissue. (B) Detailed information of biopsies. Figure S3: (A) LINE1 staining of complete tumor microarray of 48 patients with hepatocellular carcinoma, liver adenoma or focal nodular hyperplasia with corresponding normal tissues. (B) Detailed information of biopsies.
