# Supplementary material for: Combination of Antiretroviral Drugs Zidovudine and Efavirenz Impairs Tumor Growths in a Mouse Model of Cancer
Source: Viruses. 2021 Nov 30;13(12):2396. doi: 10.3390/v13122396 (PMC8703283; doi:10.3390/v13122396)
Supplement: Supplementary file 1 [file viruses-13-02396-s001.zip › viruses-1458628-supplementary.pdf]

Supplementary Figure

A

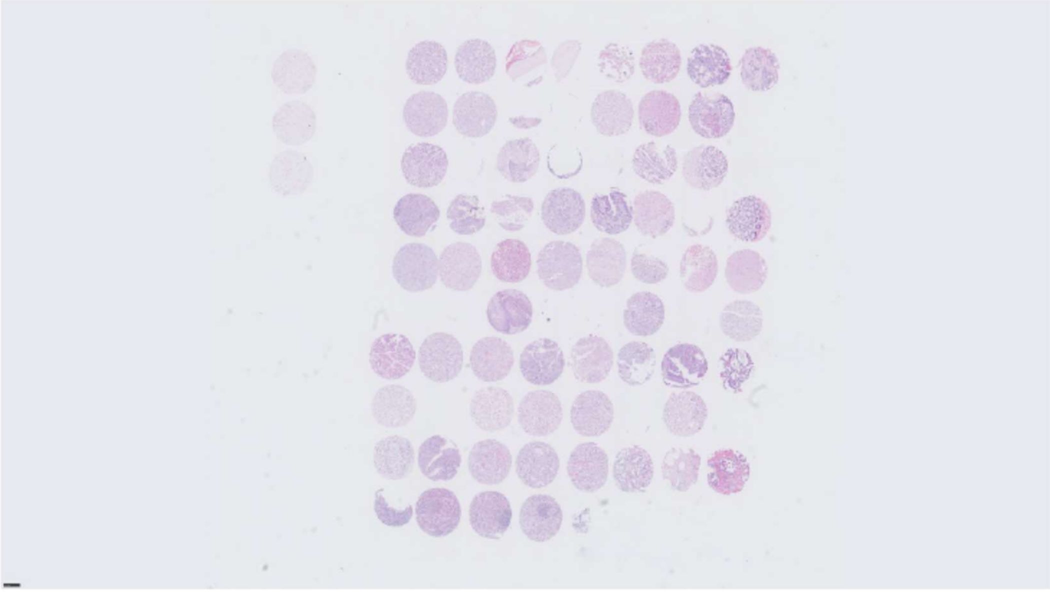

B

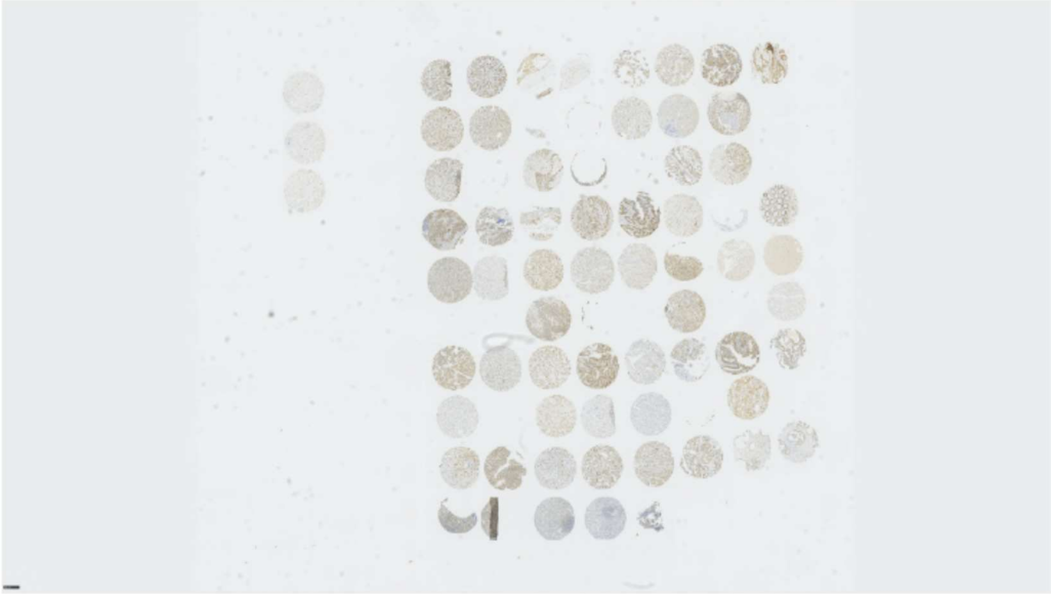

C

	1	2	3	4	5	6	7	8
y 1	Testis/ Seminom	Testis/ Seminom	Testis/ Teratom	Testis/ normal	Plazent	Plazenta	Mamma/ Inv.Duct.Ca	Mamma/ Inv.Duct.Ca
y 2	Mamma/ Inv.Duct.Ca	Mamma/ Inv.lob.Ca	Liver/ HCC	Liver/ HCC	Liver/ Cholangioca	Liver/ normal	Lung/ Adenoca	Lung/ Adenoca
y 3	Lung/ Adenoca	Lung/ Adenoca	Lung/ PlattenepithelCA	Lung/ SCLC	Pancreas/ normal	Pancreas/ Neuroendok.Ca	Pancreas/ Duct.Adenoca	Pancreas/ Anaplastisches Ca
marking y 4	Ovary/ seröses Ca	Ovary/ endom.Ca	Ovary/ muzinöses Ca	Ovary/ seröses Ca	Colon/ AdenoCa	Colon/ AdenoCa	Colon/ muzinöses Adeno	Colon/ normale mucosa
y 5	GIST/ CD117	Leiomyosarkom	Leiomyosarkom	Leiomyosarkom	Rhabdomyosarkom	Brain/ Glioblastom	Brain/ Astrozytom	Brain/ Hypocampus
y 6	Portio/ normal	Skin/ Plattenepit	Skin/ Plattenepit	Skin/MerkelzellCA	Skin/Melanom	LN/Melanommeta	PanMelanom Testblock	PanMelanom Testblock
y 7	Thyroid/ pap.Ca	Thyroid/ anapl.Ca	Thyroid/ Foll. Ca	Uterus/ AdenoCa	Uterus/ AdenoCa	Uterus/ AdenoCa	Uterus/ AdenoCa	Uterus/ normal
y 8	ccRCC	ccRCC	ccRCC	ccRCC	ccRCC	pap.RCC	kidney/ normal	kidney/ normal
y 9	UrothelCa	UrothelCa	UrothelCa	PCA	PCA	PCA	BPH	BPH
y 10	Tonsille/ normal	spleen/ Marginalzonenlymphom	spleen/normal	spleen/normal	LN/ HodgkinLymphom	LN/Mantelzelllymphom		

Supplementary Figure S1: (A) H&E staining of complete tumor microarray of 78 different tumor entities. (B) LINE1 staining of complete tumor microarray of different tumor entities. (C) Detailed information of tumor entities.

A

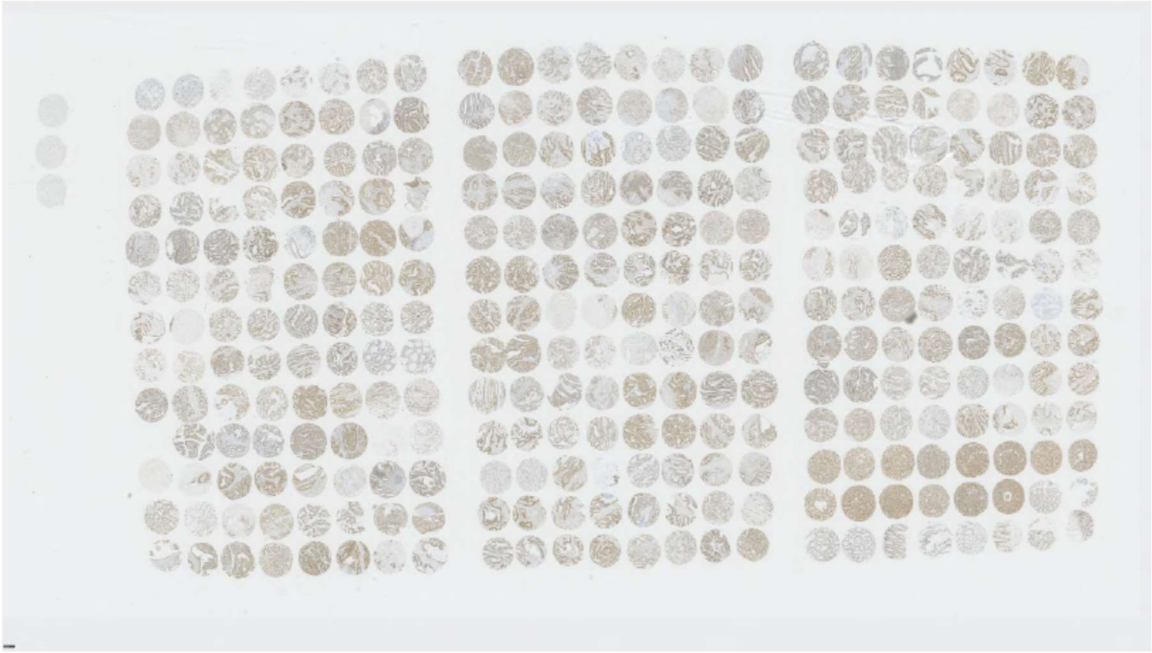

B

		x1	x2	x3	x4	x5	x6	x7	x8
	y 1	1	2	3	4	5	6	7	8
	y 2	9	10	11	12	13	14	15	16
	y 3	17	18	19	20	21	22	23	24
marking	y 4	25	26	27	28	29	30	31	32
	y 5	33	34	35	36	37	38	39	40
	y 6	41	42	43	44	45	46	47	48
	y 7	49	50	51	52	53	54	55	56
	y 8	57	58	59	60	61	62	63	64
	y 9	65	66	67	68	69	70	71	72
	y 10	73	74	75	76	77	78	79	80
	y 11	81	82	83	84	85	86	87	88
	y 12	89	90	91	92	93	94	95	96
	y 13	97	98	99	100	101	102	103	104

	x1	x2	x3	x4	x5	x6	x7	x8
y 1	105	106	107	108	109	110	111	112
y 2	113	114	115	116	117	118	119	120
y 3	121	122	123	124	125	126	127	128
y 4	129	130	131	132	133	134	135	136
y 5	137	138	139	140	141	142	143	144
y 6	145	146	147	148	149	150	151	152
y 7	153	154	155	156	157	158	159	160
y 8	161	162	163	164	165	166	167	168
y 9	169	170	171	172	173	174	175	176
y 10	177	178	179	180	181	182	183	184
y 11	185	186	187	188	189	190	191	192
y 12	193	194	195	196	197	198	199	200
y 13	201	202	203	204	205	206	207	208

	x1	x2	x3	x4	x5	x6	x7	x8
y 1	209	210	211	212	213	214	215	216
y 2	217	218	219	220	221	222	223	224
y 3	225	226	227	228	229	230	231	232
y 4	233	234	235	236	237	238	239	240
y 5	241	242	243	244	245	246	247	248
y 6	249	250	251	252	253	254	255	256
y 7	257	258	259	260	261	262	263	264
y 8	265	266	267	268	269	270	271	272
y 9	273	274	275	276	277	278	279	280
y 10	281	282	283	284	285	286	287	288
y 11	289	290	291	292	293	294	295	296
y 12	297	298	299	300	301	302	303	304
y 13	305	306	307	308	309	310	311	312

(orange)	Colorectal Carcinoma
(light)	Colorectal cancer cell lines
(yellow)	Normal colonic tissue

Nr	Colorectal cancer cell lines
289	VACO Braf V600E
290	KM12
291	SW620
292	RKO Braf V600E
293	HCT 116
294	Colo 205
295	HT 29
296	LoVo
297	CO115
298	HT55
299	VACO Braf wild-type
300	HCT15
301	LS 411 N
302	RKO Braf wild type

Supplementary Figure S2. (A) LINE1 staining of complete tumor microarray of 144 patients with colorectal carcinoma, colorectal cell lines and normal colonic tissue. (B) Detailed information of biopsies.

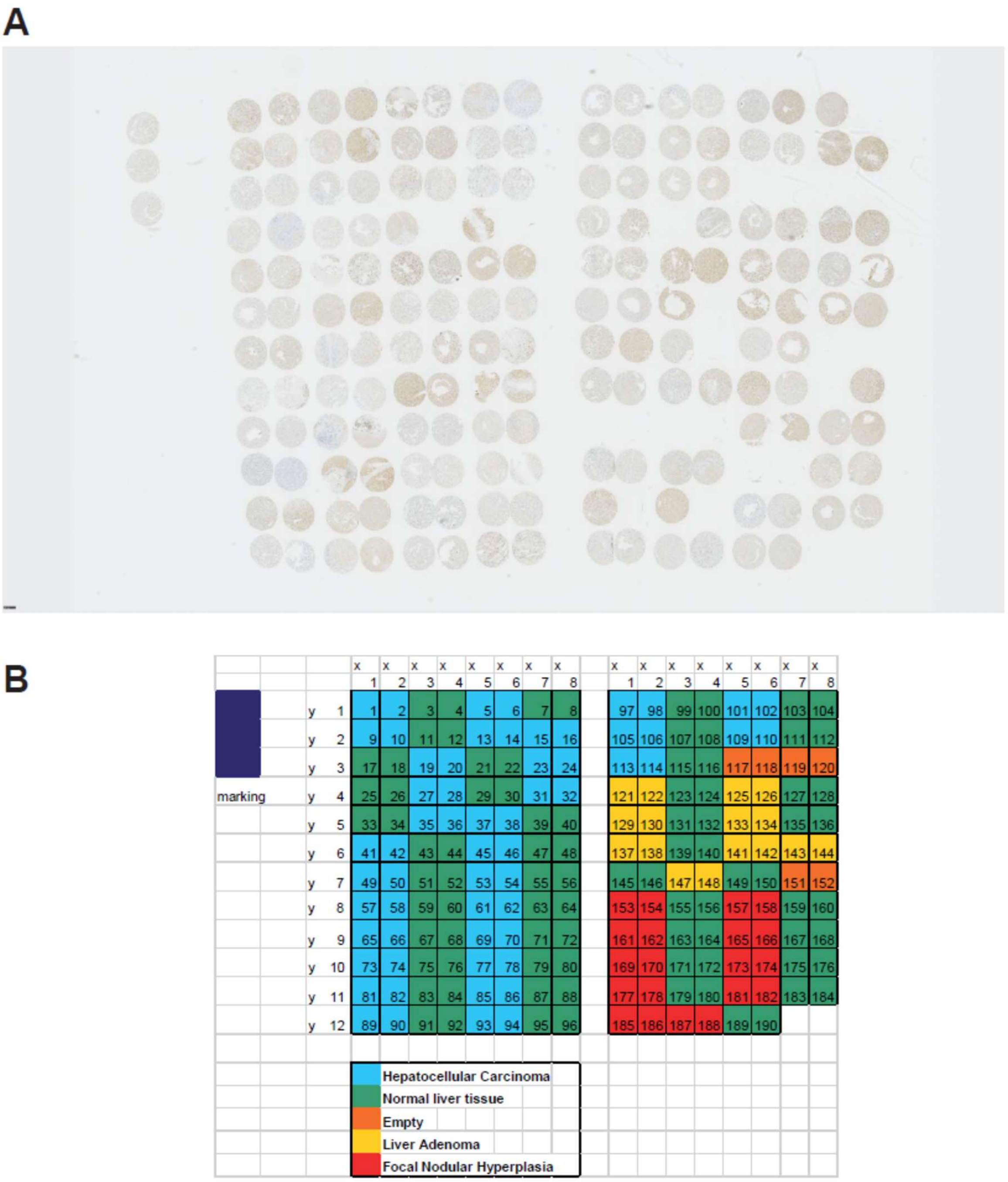

Supplementary Figure S3. (A) LINE1 staining of complete tumor microarray of 48 patients with hepatocellular carcinoma, liver adenoma or focal nodular hyperplasia with corresponding normal tissues. (B) Detailed information of biopsies.
